# Expected resolution limits of x-ray free-electron laser single-particle imaging for realistic source and detector properties

**DOI:** 10.1063/4.0000169

**Published:** 2022-11-16

**Authors:** Juncheng E, Y. Kim, J. Bielecki, M. Sikorski, R. de Wijn, C. Fortmann-Grote, J. Sztuk-Dambietz, J. C. P. Koliyadu, R. Letrun, H. J. Kirkwood, T. Sato, R. Bean, A. P. Mancuso, C. Kim

**Affiliations:** 1 European XFEL, Holzkoppel 4, 22869 Schenefeld, Germany; 2 Max Planck Institute for Evolutionary Biology, August-Thienemann-Straße 2, 24306 Plön, Germany; 3 Department of Chemistry and Physics, La Trobe Institute for Molecular Science, La Trobe University, Melbourne, Victoria 3086, Australia

## Abstract

The unprecedented intensity of x-ray free-electron laser sources has enabled single-particle x-ray diffraction imaging (SPI) of various biological specimens in both two-dimensional projection and three dimensions (3D). The potential of studying protein dynamics in their native conditions, without crystallization or chemical staining, has encouraged researchers to aim for increasingly higher resolutions with this technique. The currently achievable resolution of SPI is limited to the sub-10 nanometer range, mainly due to background effects, such as instrumental noise and parasitic scattering from the carrier gas used for sample delivery. Recent theoretical studies have quantified the effects of x-ray pulse parameters, as well as the required number of diffraction patterns to achieve a certain resolution, in a 3D reconstruction, although the effects of detector noise and the random particle orientation in each diffraction snapshot were not taken into account. In this work, we show these shortcomings and address limitations on achievable image resolution imposed by the adaptive gain integrating pixel detector noise.

## I. INTRODUCTION

Hard x-ray free-electron laser (XFEL) facilities^1–5^ have been developed and have started their operations to explore ultrafast dynamics in various science fields, e.g., biology, chemistry, physics, etc.^6–11^ Their ultrashort x-ray pulses with incomparable brightness allow one to trace electronic behaviors on the femto and picosecond timescales, before radiation damage occurs. Such measurement modes are named the “diffraction-before-destruction” method.^12^ One of the most active research areas in XFELs is serial-femtosecond crystallography (SFX), as this technique can determine structural changes of biomolecules with near-atomic resolution.^6,13,14^ However, the samples have to be crystallized for SFX, which is not always possible. As an alternative and more ambitious technique of biological structure refinement using XFELs, single-particle x-ray diffraction imaging (SPI)^15–17^ has emerged and has been applied to imaging various biological specimens, e.g., cells, cell organelles, and viruses, both in two-dimensional (2D) projection and full three dimension (3D).^18–20^

One of the biggest advantages of SPI is the potential to study protein dynamics in their native conditions, without crystallization or chemical staining. The currently achievable resolution of SPI, however, is limited to the sub-10 nanometer range. This is mainly due to instrumental limitations, e.g., pointing stability, x-ray intensity fluctuations, detector noise, and parasitic scattering from the carrier gas used in the sample delivery.^17,20–23^ Other obstacles to reach higher resolution are the heterogeneity of samples,^24^ weak scattering from biomolecules, and the effects of surrounding sample solvent.^25^ Enhancing the spatial resolution in bio-imaging by employing strongly scattering reference objects^26–33^ has been discussed and addressed as an alternative option; however, it is non-trivial to apply to 3D SPI experiments.^26^ To improve the spatial resolution of SPI at XFELs, single frame signal to background must be improved. This could include higher x-ray pulse energies with even shorter pulse durations (∼5 fs) supported by a greater number of 2D snapshots.^34,35^

The European x-ray Free-Electron Laser Facility (EuXFEL) is the first hard XFEL operating at a MHz intra-train repetition rate, supported by superconducting linear accelerator (linac) technology.^4,36,37^ Comparing to the low repetition rate around 100 Hz, the high repetition rate enables one to measure many more 2D snapshots during the SPI (and many other) experiments per unit time, although it also demands new requirements, such as commensurately high repetition rate x-ray detectors and sample delivery systems.^38–40^ Nevertheless, this high repetition rate with high pulse energies, as is now standard at EuXFEL, opens up a new era of SPI experiments simply by providing enough data for meaningful analysis in a reasonable period of experiment time. With the high repetition rate capability, more than 10 × 10^6^ frames with a half-million hits (∼5% hit rate) of iridium(III) chloride, cesium iodide, mimivirus, and Melbourne virus samples were measured during five 12 h shifts, which include instrument optimization, e.g., beamline alignment and a beam stability check.^41^ For non-biological samples, the hit rate can reach more than 10%, which enables the collection of larger datasets for SPI due to their larger x-ray cross section.^42^

To address the demand for improvements in biological SPI toward improved resolution, various simulation works and experiments have been performed to explore the possibilities. Östlin *et al.*^34^ discussed the effect of the radiation damage to diffraction signals and suggested short and intense x-ray pulses for successful SPI experiments. Fortmann-Grote *et al.*^43^ investigated the optimal x-ray pulse duration for SPI experiments for realistic pulse energies. Poudyal *et al.*^35^ described a required number of snapshots to achieve a given resolution in SPI experiments. Also, Giewekemeyer *et al.*^44^ demonstrated a successful SPI of a weakly scattering object and showed the importance of the signal-to-noise ratio level. Most significantly, and perhaps least modeled to date, detector noise and background scattering from injector systems and x-ray optics presently appear to be the key limiting factors to successful biological SPI.^41^ However, the effects of detector noise and background scatterings are not taken into account in these previous works.

In this paper, we look into how the performance of the detector sets the resolution limits of typical SPI studies of a biological system. Our work is based on the performance of the MHz detector routinely used at the hard x-ray instruments at the EuXFEL. In the standard configuration, the detector provides a single photon resolution along with a dynamic range of more than 10^3^ 12-keV photons per pixel. In the case of measurements being extremely sensitive to the noise level, the user can select a special operation mode (so-called “low-noise configuration”), which offers improved noise for the cost of dynamic range reduced to a few tens of photons. In this work, we look into both scenarios. With the help of these simulations, we study the relation between the number of diffraction patterns and the results of orientation recovery and phase retrieval in a modeled SPI experiment. We confirm that an improved separation between the noise and the one-photon peaks, in the low-noise configuration, has a tremendous impact on the SPI resolution. Adding more frames does not improve the phasing results after approaching a certain number of snapshots, due to the small but tangible presence of the detector noise.

## II. METHODOLOGY

The single particles, clusters, and biomolecules & serial femtosecond crystallography (SPB/SFX) instrument^45^ is one of the hard x-ray instrument at the European XFEL.^4^ The SPB/SFX instrument is devoted, mainly, to protein crystallography and single-particle imaging. The MHz operation of the instrument is enabled by the Adaptive Gain Integrating Pixel Detector (AGIPD).^38^ Sixteen 512×128 pixels AGIPD front-end-modules form a 1 megapixel detector, which is permanently installed at the SPB/SFX instrument. Each pixel is equipped with analogue memory cells, which allows acquisition up to 352 diffraction patterns per each pulse train (10 trains/s). As a part of the operation, re-calibration of the detector is conducted on regular basis. Typically, pedestals and noise are evaluated periodically every few hours or every time the configuration of the detector changes.

**FIG. 1.**
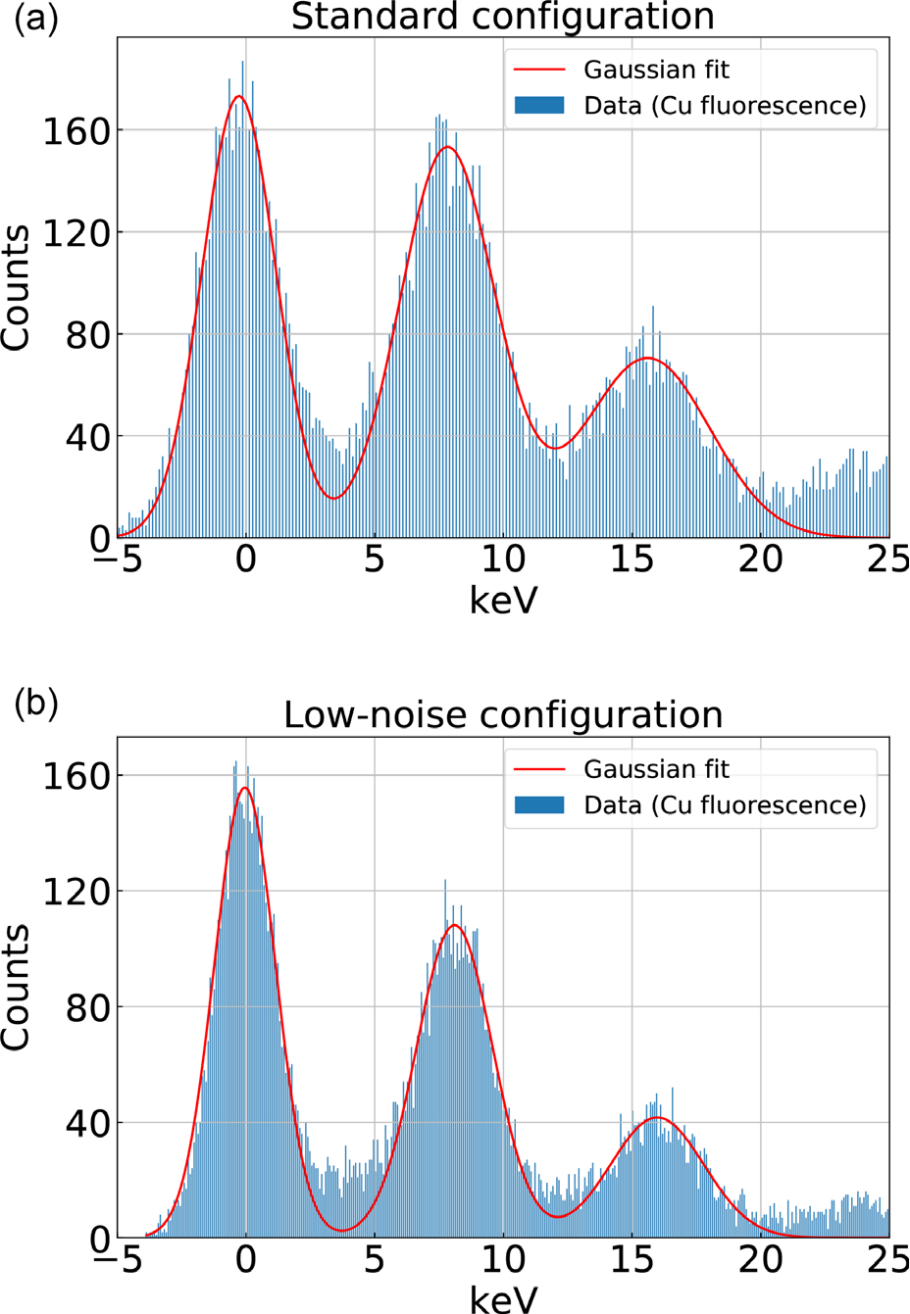
(a) and (b) The single pixel histograms of x-ray Cu $K_{\alpha }$ fluorescence collected using the AGIPD detector with two different settings: (a) standard configuration and (b) low noise configuration, at the SPB/SFX instrument. Each piece of data consists of ∼18 000 samples, and the memory cell number 2 was utilized for this measurement. The Gaussian fits of the noise, one-photon [(a) 8.09 ± 0.07 and (b) 8.15 ± 0.06 keV], and two-photon [(a) 15.88 ± 0.13 and (b) 16.02 ± 0.12 keV] peaks are represented by the solid line.

Performance of the AGIPD detector in the low count rate regime is presented in Fig. 1 showing histograms of the x-ray fluorescence from a 25-*μ*m-thick Cu foil at two different AGIPD settings (the standard configuration and the low noise configuration). The histograms are calculated from ∼18 000 samples recorded by a single pixel of the uniformly illuminated detector. Positions of one- and two-photons peaks at 8.09 ± 0.07 keV (8.15 ± 0.06 keV) and 15.88 ± 0.13 keV (16.02 ± 0.12 keV) correspond to the Cu $K_{\alpha }$ radiation with the standard (the low noise) configuration. The width of the noise peak σ=1.44± 0.08 keV (1.19 ± 0.04 keV) together with the widths of one- and two-photon peaks served as the noise estimation for the simulations.

We selected the ribosome [Protein Data Bank (PDB): 4V7V] with the antibiotics removed as a suitable model system. Its size is ∼20 nm in each dimension with ∼140 000 atoms, and the total number of electrons is ∼1 000 000. We calculated 2D diffraction patterns for realistic incident pulse energies using the “pysingfel” module^43,46,47^ integrated into the SIMEX platform.^48^ The x-ray photon energy is set to 6 keV with a pulse energy of 4 mJ, all of which is available experimentally today and typically used for SPI experiments at the SPB/SFX instrument. We assume 38% optics efficiency and 250 × 150 nm^2^ focus size (FWHM) in our calculation, which gives a fluence of $4.2\times 10^{21}$ photons/cm^2^ on the sample. The radiation damage caused by the x-ray pulse and shot-to-shot x-ray intensity fluctuations are ignored in our calculation.

**FIG. 2.**
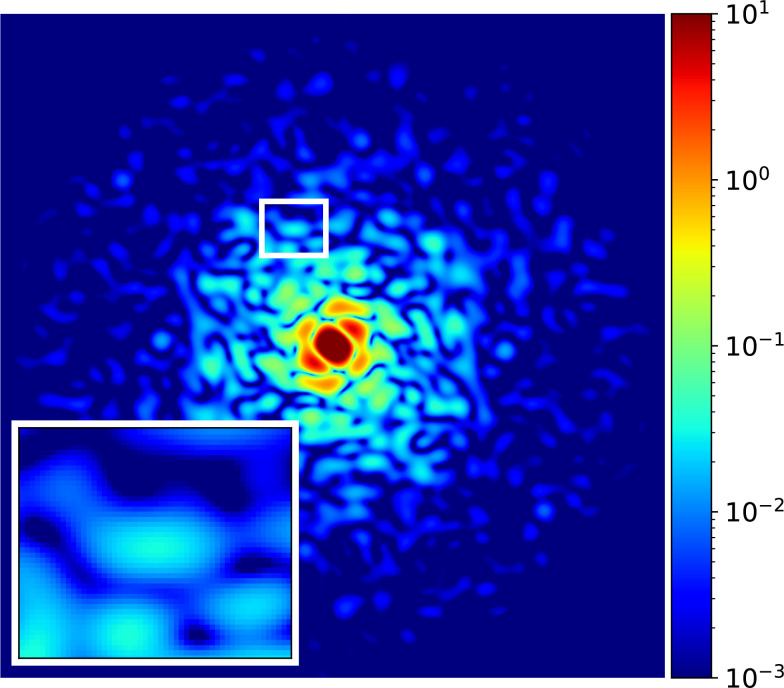
The central XY slice of the ideal reciprocal space map. The color map is coded with an arbitrary unit. The full-period resolution at the edge is 1.2 nm.

The sample-to-detector distance (SDD) is fixed to 0.25 m, and the detector pixel size is set to 200 *μ*m—the same as the AGIPD pixel size. In this configuration, the oversampling ratio ( $\frac{\text{SDD}\times \lambda }{\text{detector}\;\text{pixel}\;\text{size}}$/L) is about 12.9 in one dimension. Here, *λ* and L correspond to the x-ray wavelength and sample size, respectively. We fix the resolution at the edge of the detector to 1 nm (full-period resolution), and a 550 × 550 array (about a quarter of the total AGIPD array) is used accordingly. With a full AGIPD array, 0.5 nm, a full-period resolution at the edge of the detector is reachable in the same instrument configuration. The central XY slice of the ideal 3D reciprocal space map is shown in Fig. 2.

For a given electronic configuration, the coherent (elastic) scattering signal from bound electrons is calculated with the following equation:^43,47^

$$n(q)=\Omega \frac{d\sigma_{T}(\theta )}{d\Omega }\sum_{i}n_{\text{in}}(t_{i})|F(q,t_{i}){|}^{2},$$ (1)

where **q** is the scattering vector, Ω is the solid angle spanned by the respective detector pixel pointed by the corresponding vector **q**, $d\sigma_{T}(\theta )/d\Omega$ is the differential Thomson cross section, and F(q,t) is the structure factor for elastic scattering. Here, n(q) corresponds to the number of photons, and averaged number of photons per pixel in each single-shot diffraction pattern is about 0.09 photons (see Fig. S1). We generate each diffraction pattern with a random orientation following a uniform distribution over the SO(3) rotation group^25,43,47^ and convert the number of photons to keV units. Then, the Poisson noise is added to diffraction patterns first, and the two AGIPD detector noises are independently added by recalculating the value of each pixel, assuming that each pixel follows a Gaussian distribution scaled to 6 keV, similar to that in the histograms (Fig. 1). The scaled position of the one-photon peak is 6.07 and 6.11 keV for the studied configurations. The *σ* of Gaussian distribution for multiple photons is obtained by linear fitting of the *σ* of zero-, one-, and two-photon peaks. $\sigma_{\text{avg}}$ of the fitted *σ* values is 1.49 keV for the standard setting and 1.13 keV for the low noise setting. After applying noises, each 2D diffraction pattern is converted back to the number of photons, based on the peak separation in between zero- and one-photon peaks. In other words, signals less than 0.5 photons are set to 0 photons and signals between 0.5 and 1.5 photons are set to 1 photon. In each of the three cases (Poisson only, standard configuration, and low noise configuration), six different diffraction datasets with 5000, 10 000, 20 000, 40 000, 60 000, and 120 000 snapshots are generated with the circular beamstop area of a 5-pixel radius in the diffraction center. The circular beamstop size corresponds to the about 2 mm central gap of the AGIPD detector. The missing data regions, except the circular beamstop of the AGIPD detector, are ignored since their effect is negligible when we assemble 2D images into 3D.

**FIG. 3.**
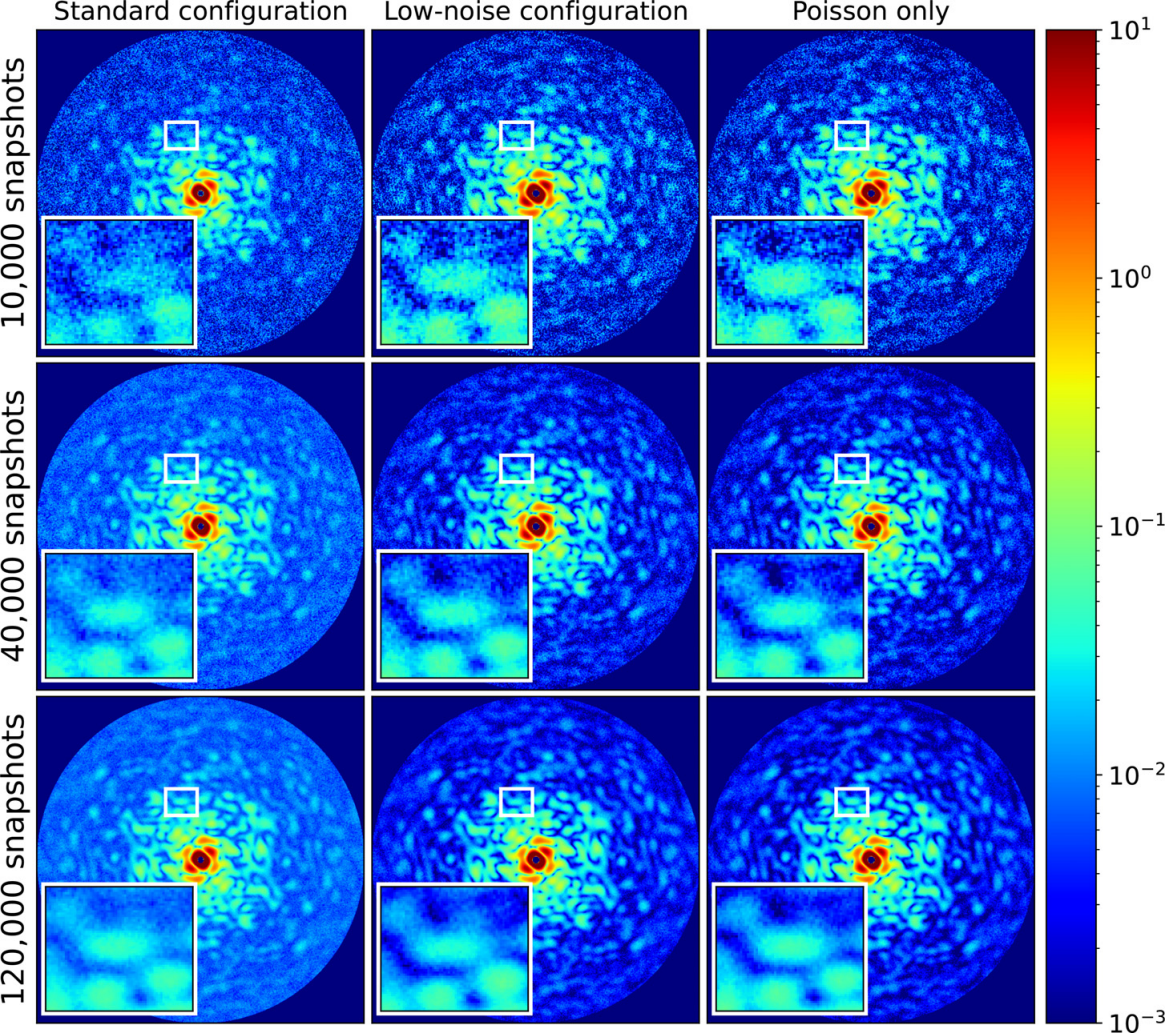
The central slices of the reciprocal space assembled using the EMC algorithm from the datasets of 10 000, 40 000, and 120 000 snapshots, respectively. The reciprocal space slices in the first and second columns are with both Poisson and AGIPD noise of the standard AGIPD configuration (first column) and the low noise AGIPD configuration (second column), while those in the rightmost column are with Poisson noise only. The color map is coded with an arbitrary unit. The full-period resolution at the edge is 1.2 nm.

We assemble the 3D reciprocal space information from the 2D frames using Dragonfly software,^49^ which was developed based on the expand–maximize–compress (EMC) algorithm.^50^ The EMC algorithm starts from a guessed “model” with random numbers, expands the model into known tomograms, uses expectation-maximization to cluster diffraction patterns into these tomograms, and compresses these “maximized” tomograms into a new model as an iteration.^50,51^ An initial 200 iterations are started with a coarse rotation group (number of rotations: 10 860) to reconstruct low-resolution (low-*q*) speckles properly. At the same time, a regularization factor, *β*, starts from 0.001 and gradually increases by factor of $\sqrt{2}$ every ten iterations. This is a regularization through deterministic annealing.^49^ This process can minimize the propensity for erratic updates, and the speckle features can be sharpened as the *β* value rises to unity. Then, we gradually increase the number of rotations available in the reconstruction's space for the data to be sorted into—36 540, 86 520, 168 900, 343 140, and 691 440—every 20 iterations to sharpen high-*q* speckles. After 300 iterations in total, the EMC reconstructed 3D grid shape is 951^3^ with the voxel size Δq=0.000 307 438 Å^−1^. The central XY slices of the EMC reconstructions of 10 000, 40 000, and 120 000 snapshots are shown in Fig. 3.

One of the EMC results is set as a reference, and the other EMC results and the ideal reciprocal space map are rotated based on the reference. The 3D reciprocal spaces resulting from the EMC reconstructions is trimmed to 552^3^ voxels after being rotated to the same orientation and binned by 2×2×2. The final reciprocal space array for phase retrieval is 276×276×276. After that, the spherical beamstop volume of 5-voxel radius from its center is masked out to remove incorrect information caused by interpolation artifacts and to keep the volume of beamstop and the ratio of beamstop over the 0th speckle the same for all datasets. Also, exactly the same spherical support, instead of a shrinking support, is used during the image reconstruction to avoid additional effects potentially caused by different shapes of a final, “shrink-wrapped” support in each reconstruction. The phase retrieval is conducted with 300 different random initial conditions, and each reconstruction is performed with 3000 iterations using the hybrid input–output (HIO) algorithm^52,53^ (for more details about the phase retrieval processes, refer to the supplementary material, Sec. III).^59^

## III. RESULTS AND DISCUSSION

After rotating the reciprocal spaces to the same orientation, slices in the middle of the Z axis are plotted from the reciprocal space of the ideal data and that of the EMC results with a different number of snapshots and detector noises. The slices are drawn in Figs. 2 and 3, respectively (for the plots of the other numbers of snapshots, refer to the supplementary material).^59^ In Fig. 3, the noise in the reconstructed reciprocal space decreases as the number of snapshots increases, especially in the higher-*q* region. In the images of 10 000 snapshots, the region in the rectangle box (Fig. 2 inset) has the poorest speckle visibility in the standard configuration. As the number of snapshots increases, the difference between the standard configuration case and the other two cases decreases. In the images of 40 000 snapshots, the difference between the standard configuration case and the others is reduced markedly, especially in the low-*q* region. However, there still exist some parts in the high-*q* region that do not match well with the ideal slice, and the overall background level is higher in the standard configuration case. When the number of snapshots increases to 120 000, all cases improve from the signal-to-noise ratio point of view; however, the standard configuration case shows more blurred details, e.g., shapes and sizes of speckles in Fig. 2 insets, especially in the high-*q* region due to the effect of the AGIPD noise on the EMC reconstruction.

The residual factor R has been used as a metric to assess the quality of diffraction patterns.^12,25,54^ We calculate the R factor, which is defined in 3D reciprocal space as Eq. (2),^55^ for the EMC reconstruction results after rotating them to the same orientation,

$$R(D)=\sum_{q\leq 2\pi /D}|\frac{\sqrt{I(q)}}{\sum_{q\prime \leq 2\pi /D}\sqrt{I(q\prime )}}-\frac{\sqrt{I_{\mathrm{ideal}}(q)}}{\sum_{q\prime \leq 2\pi /D}\sqrt{I_{\mathrm{ideal}}(q\prime )})}|,$$ (2)

where I(q) is the intensity at a scattering vector **q**, and $I_{\mathrm{ideal}}(q)$ is the intensity from the reference (undamaged) sample without any additional noise. $q=\frac{4\pi \;\text{sin}\;\theta }{\lambda }$, where *θ* is half of the incident angle 2θ between the direct and scattering x-ray, and *λ* is the x-ray wavelength. The parameter *D* is the full-period resolution.

**FIG. 4.**
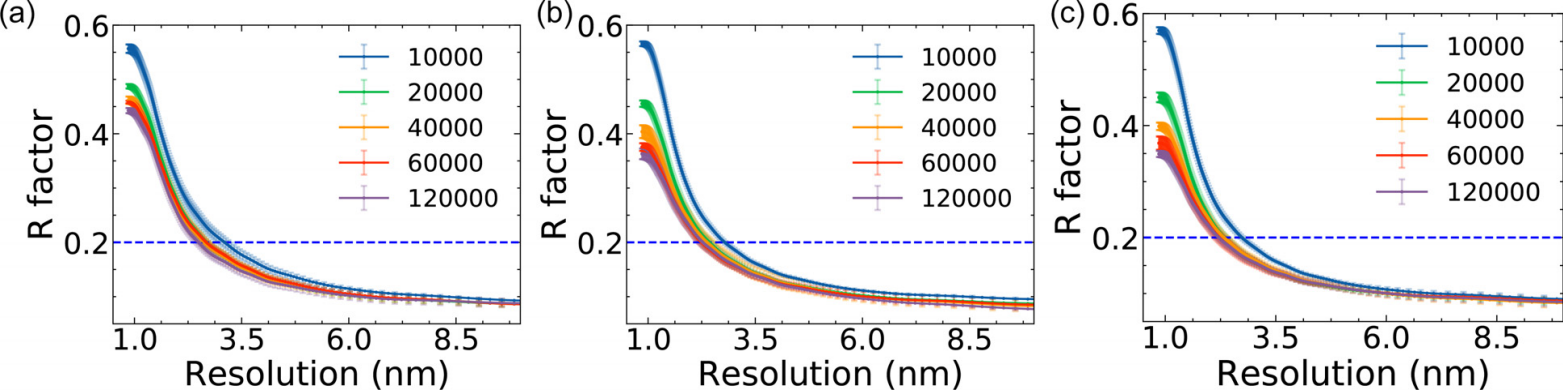
The R factor of the EMC reconstruction from the diffraction datasets with (a) Poisson + standard configuration AGIPD noise, (b) Poisson + low-noise configuration AGIPD noise, and (c) only Poisson noise, with various number (10 000–120 000) of snapshots.

The R factor results (Fig. 4) show the effects of the Poisson noise and the AGIPD noise in diffraction signal after EMC reconstruction. When there is only Poisson noise, the R factor deceases with the increasing number of snapshots [Fig. 4(c)]. In the high-resolution region where the resolution is ≲2 nm, the R factor decreases remarkably from ∼0.57 to ∼0.35, while the decrease in the R factor slows down as the number of snapshots gets larger than 60 000. In the low-resolution region (*D* > 2 nm), there is a slight improvement of the R factor between 2 and 5 nm and no longer for *D* > 5 nm. The trend of R factor shows that increasing the number of snapshots can help increase the quality of diffraction patterns, especially in the high-resolution region, which is desired in the experiment.

In the case of the standard configuration, the R factor decreases as the number of snapshots increases; however, the R factor is much higher than that with only Poisson noise, which indicates the achievable resolution in the standard configuration is much worse than the Poisson-only case with the same number of snapshots. In the higher resolution region where the resolution is ≲2 nm, the R factor decreases from ∼ 0.55 to ∼ 0.45, mostly in between 10 000 and 20 000 snapshots. In the low-resolution region (*D* > 2 nm), the behavior of the R factor is almost the same as the Poisson-only case.

In the case of the detector set to the low-noise configuration, the R factor decreases as the number of snapshots increases, as for the other two cases. Due to the improved peak separation, the R factor is lower than that in the standard configuration case and similar to that with only Poisson noise. The improved signal-to-noise ratio suggests that fewer snapshots are needed to reach the same reconstruction quality as in the standard configuration, and the quality is close to the case with only Poisson noise when we compare the result with the same number of snapshots. More generally, this means that per-frame detector noise represents a practical limit to the potential resolution achievable in SPI—at least for today's methodology.

**FIG. 5.**
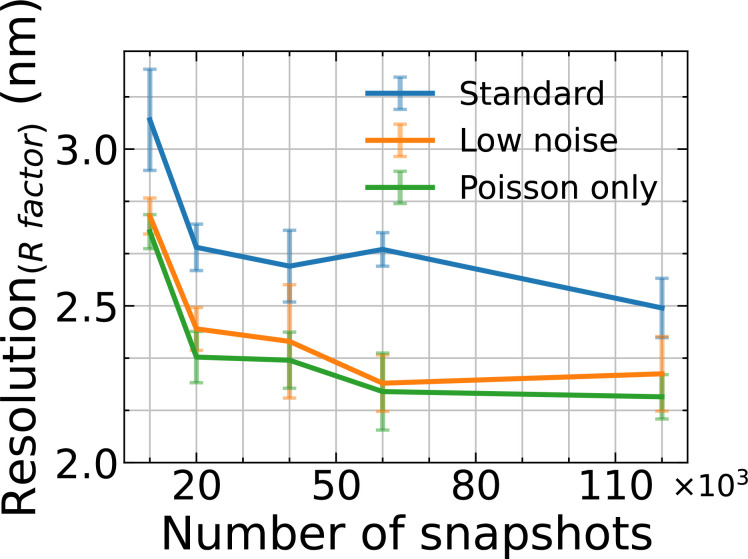
Estimated full-period resolution based on the 0.2 threshold level in the R factor analysis, as shown in Fig. 4. The error bars are the standard deviation of the resolutions estimated from multiple EMC results of the same diffraction datasets with the same EMC parameters.

During the R factor analysis, we omit the 5000 snapshots result since the noise at the high-*q* region distorts the R factor analysis when the number of snapshots is considerably insufficient (for more details about the noise analysis, refer to the supplementary material, Sec. II).^59^ The blue dotted lines in Fig. 4 correspond to 0.2 threshold, which is a typical confidence level for x-ray crystallographic data, e.g., crystal structures of biomolecules in the PDB^56^ and also used in SPI.^12,25,54^ We determine the achievable resolution by taking the point of intersection of our measured R factor with the *R* = 0.2 horizontal line. The resolution as a function of the number of snapshots is plotted in Fig. 5. As we described in the previous paragraphs, the resolution of all three cases is improved continuously as the number of snapshots increased, although the improvement slows when the number of snapshots gets larger than 20 000 for the case here. As compared with Poisson-only case, the achievable image resolution with the standard AGIPD configuration is much poorer, while it is comparable for the optimized settings.

For the further systematic analysis, the phase retrieval process is applied to the 3D reciprocal space data that have been produced by the EMC reconstructions and after orientation correction (as we describe in Sec. II). As the resolution in the standard configuration is apparently lower than that of the other two cases, we conduct phase retrieval only on the low-noise and Poisson-only cases to explore the best achievable phase retrieval result with the AGIPD detector.

**FIG. 6.**
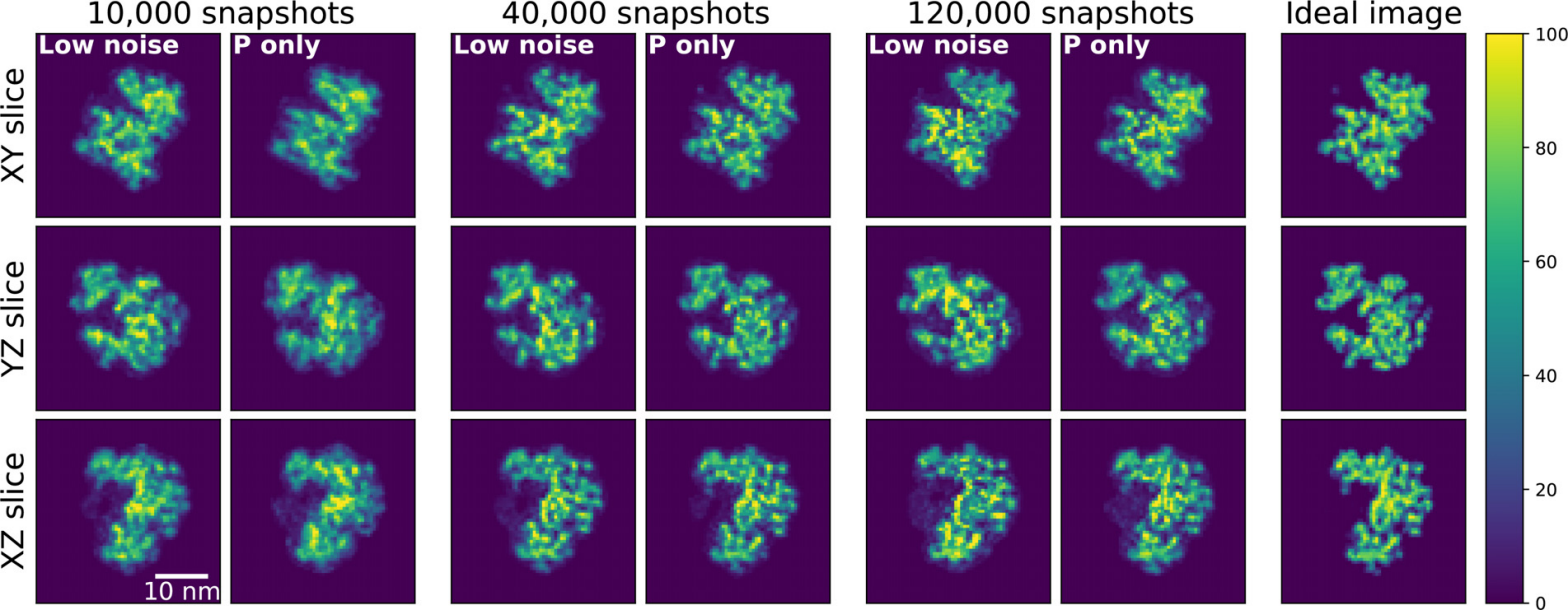
The central slices of the 3D phase retrieval real space results, which are reconstructed using the 3D reciprocal space assembled with various number of snapshots as input. The ideal real space electron density slices are plotted in the rightmost column as a reference. The unit of the color bar is the number of electrons in a voxel.

The retrieved real space images from different initial conditions are aligned by comparing the center-of-mass of the reconstructed images. The three best reconstructions, defined as those with the smallest reconstruction error in the reciprocal space, are averaged in each dataset. The smallest reconstruction error in the reciprocal space is calculated by comparing the recovered 3D reciprocal space intensity map after phase retrieval with the input EMC result. Since each dataset (different number of snapshots and noises) has a slightly different center of mass, further alignment, based on the ideal image is performed. First, a ten times bigger array size in each dimension is generated by using an interpolation method, and the center of each array is re-optimized, based on the ideal image. Afterward, a binning process is applied to return the arrays back to their original size. The overall background in the reconstructed image is rather high with 10 000 snapshots, though improves as the number of snapshots increases, again due to the improvement of the signal-to-noise ratio. To minimize the effect of the background noise caused by the spherical support in the electron density calculation, Gaussian blurring is used to refine a tighter support. After applying the tight support, we convert the unit of the final images to the number of electrons in a voxel. Figure 6 shows the central slices (XY, YZ, and XZ) of the averaged images of 10 000, 40 000, and 120 000 snapshots together with those of the ideal image. The slices of the images with other numbers of snapshots included can be found in the supplementary material.^59^

The effect of the number of snapshots is clearly observed in the reconstructed objects (Fig. 6). The overall shape and fine structures are clearer and get closer to the ideal ribosome image (Fig. 6, rightmost column), which is “ideal” and the “original” electron density converted from the PDB file. Except for the phasing result of 10 000 snapshots, the results of the Poisson-only case are always better. When the AGIPD noise is implemented, we could observe that there are artifacts in fine structures. Also, detailed structures in the upper part of the XY slice and the right side of the XZ slice of the averaged images of 40 000 snapshots are much closer to the ideal ribosome image as compared with those of 120 000 snapshots, which means that adding more snapshots without improving the detector noise can generate more artifacts, especially during the phase retrieval process. Our result indicates that the AGIPD detector behaves almost as a single photon counting detector.

## IV. CONCLUSIONS

In summary, we have addressed the effect of detector noise in EMC reconstructions and the retrieved real space images for a realistically modeled SPI experiment and a real XFEL detector, AGIPD. The overall shape information of the ribosome can be easily retrieved, even with 10 000 snapshots, though for the parameters used here, more than 20 000 snapshots are desirable to overcome the intrinsic photon counting noise and to have a sufficient signal-to-noise ratio. The improvements of the EMC reconstruction and the retrieved real space image for an increasing number of frames decreases after approaching a certain number of snapshots. In general, it is desirable to measure as many snapshots as feasible for a successful SPI experiment, though we see here that there is a ceiling on the useful number collected—more than 40 000 snapshots without improving the AGIPD detector noise for the cases shown here does not help. The achievable full-period resolution, based on the 0.2 threshold level in the R factor analysis of the ribosome sample, is ∼2.5 nm with 6 keV x-ray energy and 0.25 m sample to detector distance. In practice, the achievable resolution may be poorer if we consider the parasitic scattering from the carrier gas used in the sample delivery. This experimental effect can be improved by, e.g., changing the carrier gas from N_2_ to He, which is under development.

As shown in our results, a key aspect in reducing the influence of detector noise is to increase the separation between noise and one-photon peaks in Fig. 1. With the conditions used in the simulations here (6 keV, the standard mode and the low-noise mode),^38^ the peak separations are 4.07 $\sigma_{\text{avg}}$ and 5.41 $\sigma_{\text{avg}}$, with $\sigma_{\text{avg}}$ being the averaged standard deviation of the fitted noise, one- and two-photon peaks in each case, respectively. When we consider the standard configuration of the AGIPD detector, the EMC reconstructed 3D diffraction image quality is quite poor due to the significant overlap between neighboring peaks. However, after tuning the detector settings to the low-noise configuration, the EMC reconstructed 3D diffraction image quality is quite similar to that of the Poisson-only case although the effect of the detector noise can be seen in the retrieved real space images of 120 000 snapshots. Further increase in photon separation can be achieved linearly by increasing x-ray photon energy. This approach has to be carefully balanced with the decreased scattering cross section as the photon energy is increased. The photon separation required to eliminate the effect of detector noise in 3D single-particle reconstructions is a topic of further study. In addition to the simple analytical model used for noise generation in this paper, the detector simulation pipelines based on the x-ray Camera Simulation Toolkit (X-CSIT)^57^ developed at the EuXFEL can be implemented for more realistic detector response simulation in the future.

In conclusion, the AGIPD detector is well-suited for biomolecule SPI experiments at MHz rate at the SPB/SFX instrument. We expect that the better performance of the high repetition rate detector at the EuXFEL, improving background level caused by the sample carrier gas, and having more x-ray photon flux by optimizing the EuXFEL machine will enable us to explore various biological specimens in their native conditions at ever improving resolutions.

## Data Availability

The data that support the findings of this study are openly available in Zenodo, at https://doi.org/10.5281/zenodo.7257401, Ref. 58. Raw data were generated at the EuXFEL large scale facility. Derived data supporting the findings of this study are available from the corresponding author upon reasonable request.
